# Supplementary material for: Safety and Regenerative Properties of Immortalized Human Mesenchymal Stromal Cell Secretome
Source: Int J Mol Sci. 2025 Sep 24;26(19):9322. doi: 10.3390/ijms26199322 (PMC12525087; doi:10.3390/ijms26199322)
Supplement: Supplementary file 1 [file ijms-26-09322-s001.zip › ijms-3792649-supplementary.pdf]

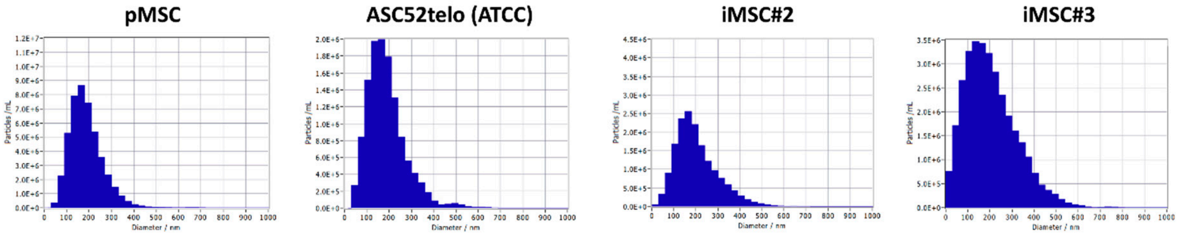

	pMSC	ASC52telo (ATCC)	iMSC#2	iMSC#3
Size on peak, nm (Mean±SD)	167.02 ± 7.06	157.43 ± 16.58	163.06 ± 4.29	158.15 ± 12.69
Concentration, paricles/ml (Mean±SD)	4.78*10^8 ± 1.46*10^8	3.94*10^8 ± 2.11*10^8	2.57*10^8 ± 1.27*10^8	3.38*10^8 ± 5.56*10^7

Figure S1. Nanoparticle Tracking Analysis (NTA) of the extracellular vesicles of primary and immortalized MSC cultures. No significant differences in size and concentration of vesicles are detected.

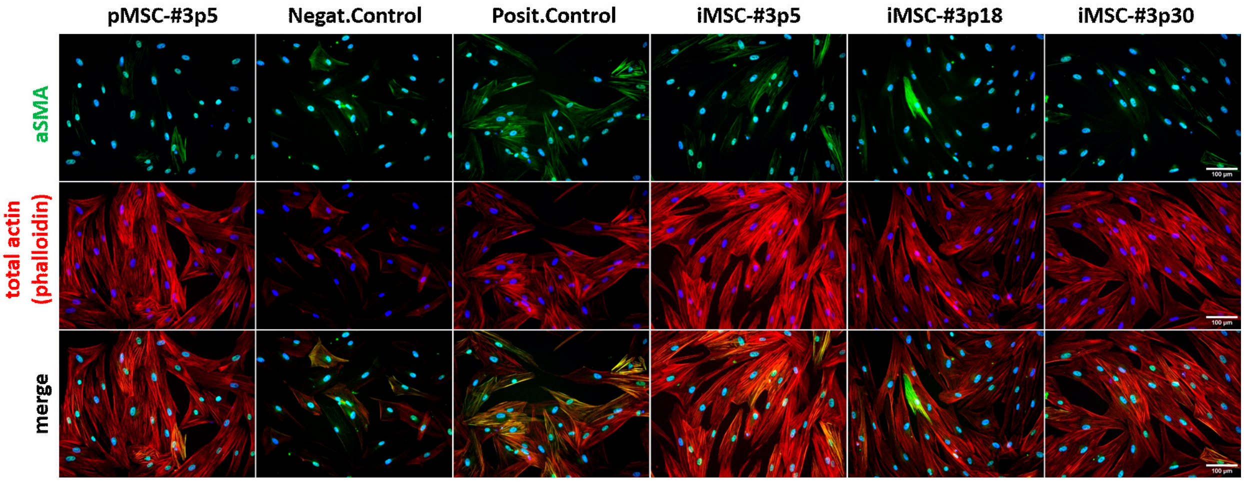

Figure S2. Separate visualization of aSMA and total actin expression (phalloidin staining) in fibroblast-myofibroblast cultures in the in vitro model of fibrosis.

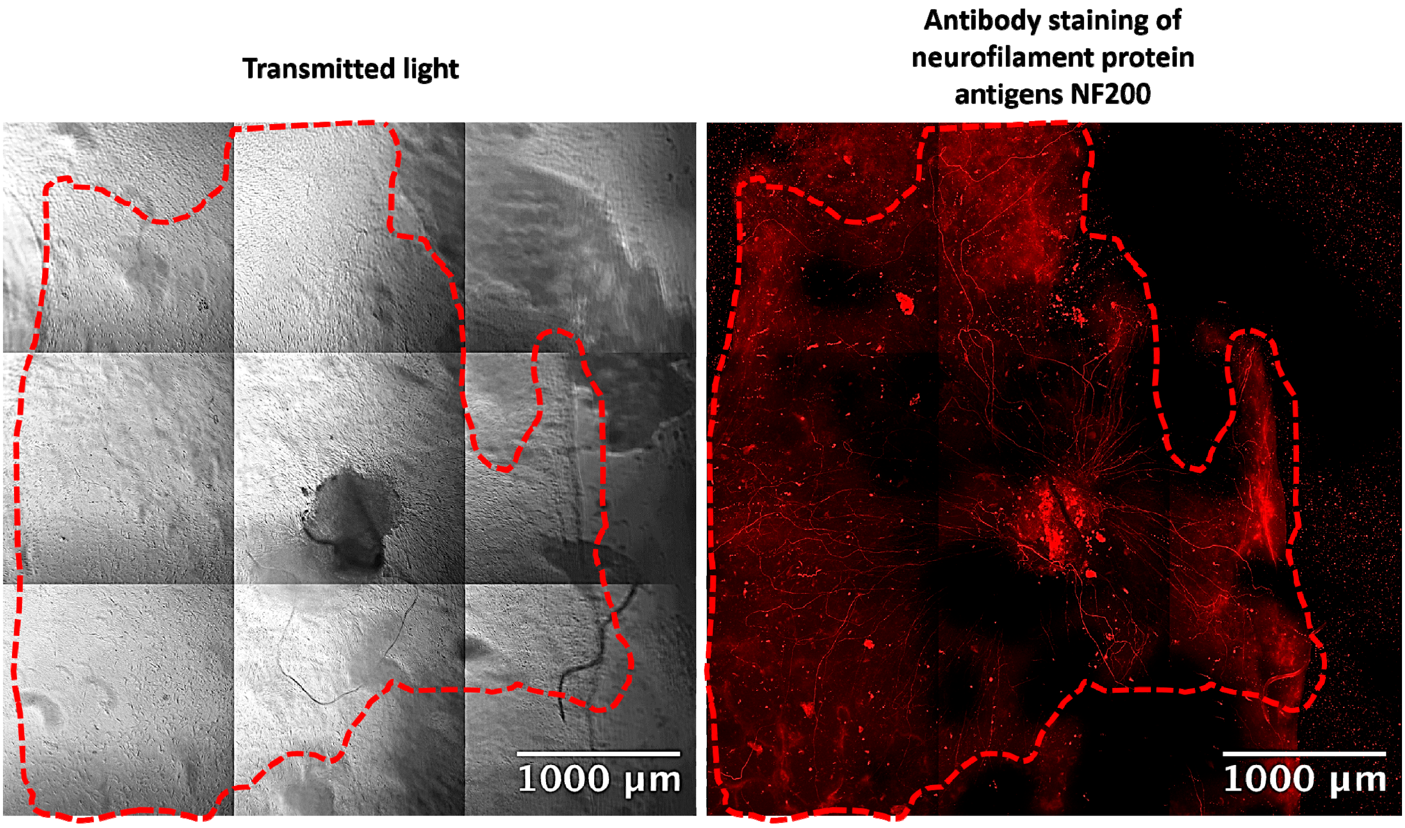

Figure S3. Examples of staining of nerve fibers of an explant culture of mouse spinal ganglia with antibodies to NF200 (red staining, Alexa Fluor™ 594). Nerve fibers are identified as thread-like structures extending from the ganglion to the periphery. The red dotted line marks the boundary of the nerve fiber growth front.

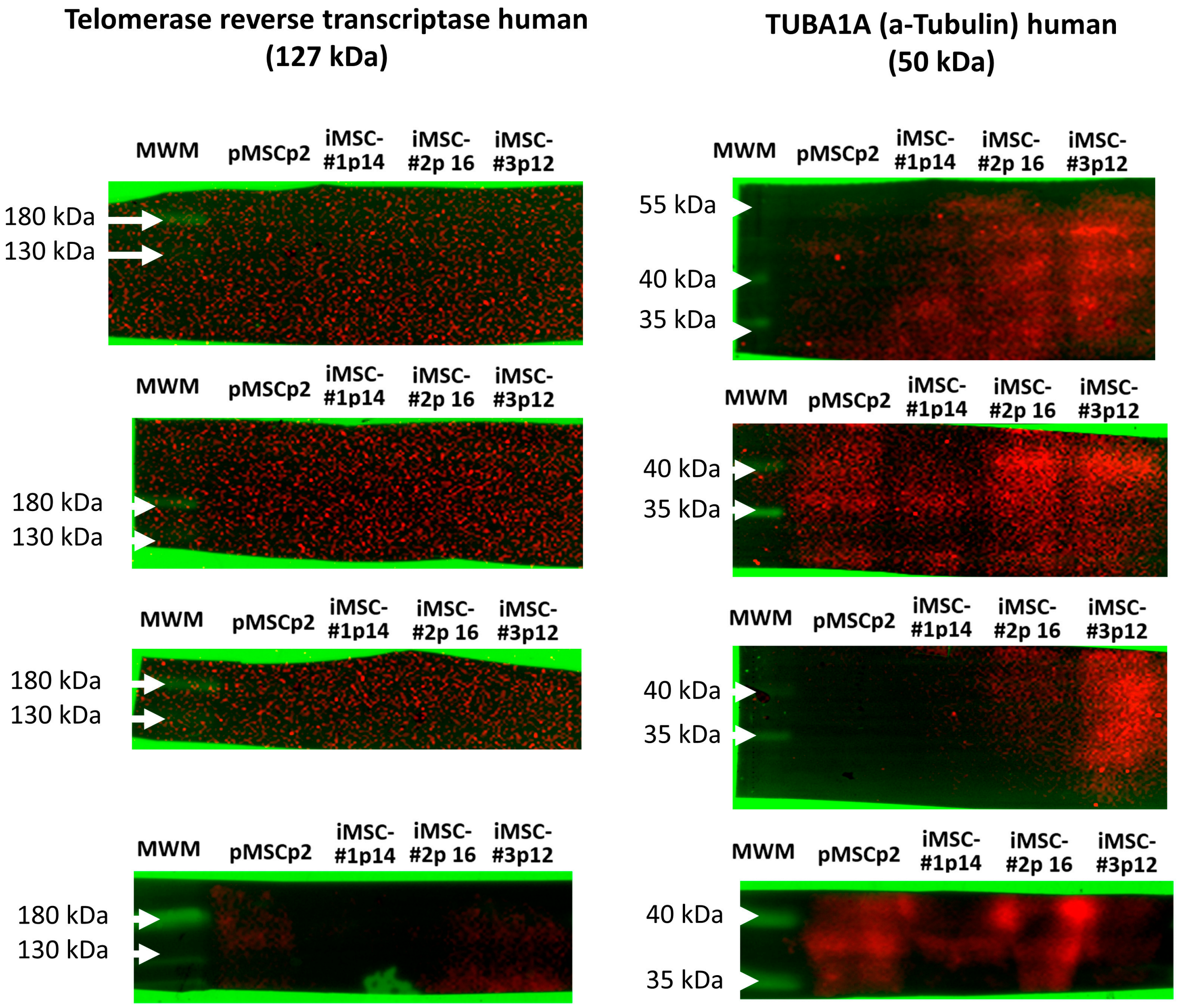

Figure S4. The results of four independent experiments on detecting the catalytic subunit of human telomerase in the 100-fold concentrated pMSC and iMSC secretome. Staining of the blot for TUBA1A demonstrates the sensitivity of the assay to detect intracellular proteins in the secretome.

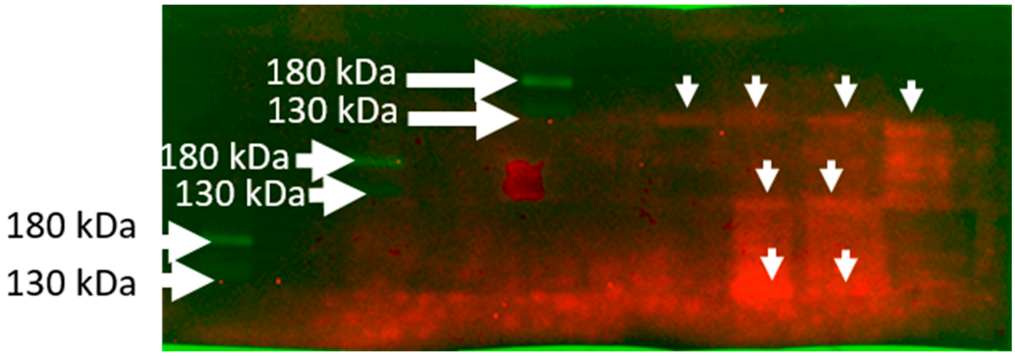

Figure S5. Evaluation of the performance of antibodies against the catalytic subunit of human telomerase hTERT using cell lysates of immortalized MSCs. This PVDF membrane accommodated several strips of different PAA-gels (arranged in three horizontal layers) containing the proteins within the range 70-180 kDa and including telomerase. White vertical arrows point to bands corresponding to telomerase by mass, confirming the performance of the antibodies and the Clarity™ Western ECL Substrate kit.

Supplementary Table S1. Primers and amplification parameters used in the study

Target mRNA	Primer Sequence (5'->3')	Annealing Temperature, ^0C*	Amplicon length, b.p.
TERT	ACCGTGGTTTCTGTGTGGTG<br>TCGCCTGAGGAGTAGAGGAA	58	211
BDNF	TTAGGGGTCAAGGTGGGCTA<br>AGGCTCCAAGGGAACTTGTG	57	236
uPA	ATTGCGACTGCCTGAATGGA<br>TCCGACCTGCACATAACACC	58	326
VEGFA	ATCAAACCTCACCAAGGCCAG<br>AGGCCCACAGGGAACGC	58.5	183
HGF	ACCACACCGGCACAAATTCT<br>ATCCCAACGCTGACATGGAAT	58	274
IL1A	AGTAGCAACCAACGGGAAGG<br>ATGTAATGCAGCAGCCGTGA	58	256
IL6	TGCAATAACCACCCCTGACC<br>ATTTGCCGAAGAGCCCTCAG	57.5	150
TNFA	CTCTCTGCCATCAAGAGCCC<br>TCCCAAAGTAGACCTGCCCA	58	181
IL4	TTTGCTGCCTCCAAGAACAC<br>GTTCCTGTCGAGCCGTTTCA	57	171
IL10	TGCTCTGTTGCCTGGTCCT<br>GTCTGGGTCTTGGTTCTCAGC	58.5	296
IL13	CATCCGCTCCTCAATCCTCTC<br>GGGTCTTCTCGATGGCACTG	58	286
IDO	CAACCCCCAGCTATCAGACG<br>GGAGGAACTGAGCAGCATGT	58	161
TGFB1	AAGTGGACATCAACGGGTTC<br>GAGGCAGAAGTTGGCATGGT	56	272
36B4	CGACCTGGAAGTCCAACTAC<br>ATCTGCTGCATCTGCTTG	53	109

* - estimated annealing temperature for PCR using mixtures containing Taq polymerases
